# A mobile system for whole eye perfusion supporting retinal function and surgery

**DOI:** 10.3389/fbioe.2025.1699876

**Published:** 2026-01-16

**Authors:** Maxwell Lohss, Katelin S. Samski, Alkiviades Liasis, Hamzah Aweidah, Chiaki Komatsu, Oliver Beale, Daniel M. W. Lee, Ethan A. Rossi, Sanjeev G. Shroff, José A. Sahel, S. Tonya Stefko, Leah C. Byrne

**Affiliations:** 1 Department of Ophthalmology, University of Pittsburgh, Pittsburgh, PA, United States; 2 Department of Radiology, University of Michigan, Ann Arbor, MI, United States; 3 Department of Plastic Surgery, University of Pittsburgh, Pittsburgh, PA, United States; 4 Department of Bioengineering, University of Pittsburgh, Pittsburgh, PA, United States; 5 McGowan Institute for Regenerative Medicine, University of Pittsburgh, Pittsburgh, PA, United States; 6 Institut De La Vision, Institute National De La Santé Et De La Recherche Médicale, Centre national de la recherche scientifique, Sorbonne Université, Paris, France; 7 Centre Hospitalier National d’Ophthalmologie des Quinze-Vingts, Paris, France; 8 Department of Otolaryngology, University of Pittsburgh, Pittsburgh, PA, United States; 9 Department of Neurological Surgery, University of Pittsburgh, Pittsburgh, PA, United States; 10 Department of Neurobiology, University of Pittsburgh, Pittsburgh, PA, United States

**Keywords:** adaptive optics scanning laser ophthalmoscope (AOSLO), electroretinography (ERG), *ex-vivo* perfused eye, fluorescein angiography, fundus, isolated perfused eye, living extracorporeal eye, optical coherence tomography (OCT)

## Abstract

**Introduction:**

The use of human donor tissue has the potential to accelerate translational research and support the development of effective human medicines. However, post-mortem sample degradation and the loss of anatomical context limits the utility of primary tissue.

**Methods:**

Here, an ex vivo perfusion platform, the Advancing Straight-to-Human Eye Research (ASTHER) system, was designed to perfuse whole eyes with autologous blood to support ex vivo viability. The system was designed for portability and compatibility with modern surgical techniques.

**Results:**

Feasibility trials of ophthalmic artery cannulation, arterial perfusion, retinal imaging, and electroretinography with the ASTHER platform were performed.

**Discussion:**

Qualitative imaging and electroretinography confirmed post-enucleation retinal blood supply and neuronal function in perfused Yucatan mini pig eyes. Retinal surgery was performed in perfused eyes. A pilot study showed the applicability of the minipig enucleation and perfusion protocol in donated human tissue. Future validation studies of the ASTHER platform will quantify perfused tissue structure and function.

## 1 Introduction

Vision impairment is a growing public health concern, currently affecting over 43 million people worldwide. This number is projected to exceed 60 million by 2050 due to aging populations and rising prevalence of retinal diseases (Bourne et al., 2021). In the United States alone, vision loss is associated with an estimated $139 billion in annual healthcare expenditures and contributes to reduced quality of life, independence, and productivity (Wittenborn et al., 2013). A large proportion of these cases stem from retinal damage, which is often irreversible. The retina’s neural and vascular structures are vulnerable to ischemia, degeneration, and trauma, all of which can result in permanent vision loss. Scientific study of retinal damage has historically relied on the use of animal models as platforms to investigate disease and develop surgical and pharmacological interventions.

Despite a well-established scientific history, animal models fall short of accurately representing human anatomy and physiology for vision research. Rodents - while widely used - differ substantially from humans in both structure and scale. They lack a macula, the high-acuity retinal region essential to human vision, and their small eye size limits surgical and imaging techniques (Winkler et al., 2020; Moshiri, 2021). These anatomical differences constrain the translational relevance of rodent-based models, particularly for techniques requiring precision vascular access or macular targeting. Larger animal species - including pigs and non-human primates (NHPs) - offer closer anatomical analogues. Pigs have long been established as a model for vascular and perfusion research (Vrselja et al., 2019). Their eyes are similar in size to human eyes and have robust, accessible vasculature, making them good candidates for surgical access and device testing (Vestergaard et al., 2019). While pigs lack a macula and fovea by human standards, their eyes contain a strip of concentrated photoreceptors and ganglion cell layers that show a high cone and rod density (Choi et al., 2021). This region is referred to as the visual streak and can serve as an approximation of a macular region but is not a true replica. The macula of NHPs is critical for studying macula-specific diseases and gene therapy approaches (Drag et al., 2023). However, their limited availability, high cost, and fine vascular anatomy pose technical barriers (Burgoyne, 2015; Picaud et al., 2019) for widespread scientific use.

Differences in species’ protein homology, immune response, and molecular signaling can alter therapeutic efficacy when treatments are translated from the bench to bedside (Brinks et al., 2011; Frangogiannis, 2022) no matter the animal model used. In the Luxturna trials, full field electroretinogram improvements observed in canine studies were not replicated in human participants, and humans experienced a decline in visual function over time not seen in animal models (Trapani and Auricchio, 2018). These inconsistencies emphasize the need for models that align not only anatomically and physiologically, but also at molecular and genetic levels.

Donated human tissues are a crucial resource in vision sciences as they are the most relevant platform for the scientific study of human anatomy and pathology. Explants of ocular tissues are commonly used to study the causes, progression, pathology, and potential treatments for many eye conditions. Retinal explants allow for the testing of gene and cell therapies, drug screening and toxicology studies, electrophysiology studies, the development of new imaging modalities, as well as the study of diseases through molecular and histological investigation (Schnichels et al., 2021; Saeid et al., 2025). Conversely, retinal explanted tissues are limited in their utility by their post-mortem degradation and the loss of anatomical context, such as the presence of the blood retinal barrier and vitreous, which make the treatment of retinal pathophysiology challenging (Ben-Arzi et al., 2022).

*Ex vivo* eye perfusion systems offer a controlled setting for maintaining and evaluating the tissues of an enucleated ocular globe. Historically, these platforms have allowed for the precise manipulation of variables such as flow rate, perfusion pressure, and intraocular pressure (Niemeyer, 1975). Prior animal models of *ex vivo* arterial eye perfusion have supported investigations into retinal metabolism, ocular pharmacology, electrophysiologic activity, and vascular dynamics (Niemeyer, 2001). To the best of the authors’ knowledge, only one study of whole eye arterial perfusion using human tissue has been published (Anderson, 1991). The tissue was acquired from two brain-dead human donors during organ procurement for transplantation. For one eye, 20 min elapsed from enucleation to cannulation, and a short, 8 mm, optic nerve segment precluded cannulation of the ophthalmic artery; instead, one posterior ciliary artery was cannulated. The second eye experienced a 42-min period of global ischemia prior to *ex vivo* perfusion; ophthalmic artery cannulation was completed. Abnormal electrophysiological signals were recorded from both eyes when perfused at 37 °C.

Unfortunately, most animal and human models of *ex vivo* perfusion majorly predate modern advances in ophthalmic imaging and intervention - such as spectral-domain optical coherence tomography (OCT), adaptive optics scanning light ophthalmoscopy (AOSLO), subretinal gene therapy, and whole eye transplantation (Drag et al., 2023; Niemeyer, 2001; Roorda et al., 2002; Fujimoto et al., 2023; Scarabosio et al., 2024). As therapeutic strategies evolve, this underscores the renewed need for versatile *ex vivo* systems in anatomically relevant models.

The Advancing Straight to Human Eye Research (ASTHER) perfusion system is a mobile platform designed to preserve the anatomical, vascular, and functional integrity of enucleated human eyes. The goals of this research were to:

1. Develop a perfusion system capable of transport from the operating room to the laboratory without any interruption of perfusion.
2. Demonstrate retinal perfusion and evaluate retinal structure using high-resolution imaging techniques.
3. Assess retinal function by recording electroretinograms in perfused eyes.
4. Maintain global anatomical integrity of the eye, including vascular architecture and ocular morphology.
5. Enable intraocular surgical procedures during perfusion.
6. Evaluate the applicability of the pig perfusion protocol in human tissue.

## 2 Methods

The following methods are organized according to the six primary objectives listed in the Introduction above. Data is reported as mean ± standard deviation unless otherwise noted. Data from experiments outside of the current scope of the manuscript or from eyes used for other protocols are not reported.

### 2.1 Develop a perfusion system capable of transport from the operating room to the laboratory without any interruption of perfusion

A single-pass *ex vivo* eye perfusion system was constructed using a MINIPULS 3 peristaltic pump (Gilson, Middleton, WI, United States) with silicone tubing and a customized eye support chamber. The system is mounted on a mobile, height-adjustable cart equipped with adapters for clinical imaging modalities, electrophysiological evaluation, and surgical instrumentation. Portable power stations (EF4, EcoFlow, Seattle, WA, United States) allow for up to 2 hours of operation during power failure and transport.

**FIGURE 1:**
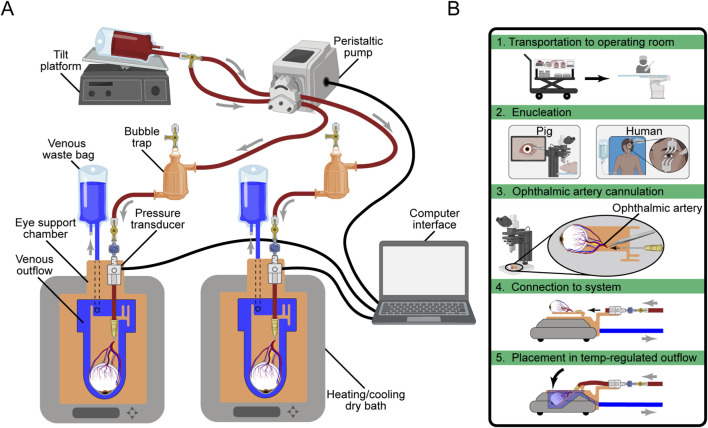
**(A)** Schematic of the ASTHER eye perfusion system. **(B)** Workflow for ocular tissue perfusion.

Volumetric flow rate and perfusion pressure are continuously recorded using a PowerLab data acquisition system and are displayed on the PowerChart user interface (ADInstruments, Dunedin, New Zealand). Pressure data was imported into MATLAB (MathWorks, Natick, MA, United States) for analysis. A 1-min moving average filter was applied to reduce signal noise. To account for venous refilling after ischemic collapse, pressure data from the first 30 min after cannula connection is excluded from averaging calculations. Blood in a standard IV bag is stored on a continuously rotating tilt platform (BlotBoy™, Benchmark Scientific, Sayreville, NY, United States), routed through a three-way stopcock, and divided into parallel tubing lines that pass through the peristaltic pump. Each circuit includes a 3D-printed bubble trap designed in SolidWorks (Dassault Systèmes, Waltham, MA, United States) and fabricated on a Form 3B stereolithography printer (Formlabs, Boston, MA, United States) using Surgical Guide resin (RS-CFG-SGAM-01, Formlabs, Boston, MA, United States). The perfusate passes through a one-way stopcock with a downstream injection port, where a pressure transducer (MLT1199, ADInstruments, Dunedin, New Zealand) monitors arterial pressure before entering the eye. Venous drainage from the eye flows out of the vortex veins, over the cannulation platform, and into an angled reservoir within the eye support chamber, where it then gravity drains into a waste collection bag. Both the eye support chamber and cannulation platform were designed in SolidWorks and 3D-printed using High Temp (RS-CFG-HTAM-02, Formlabs, Boston, MA, United States) and Surgical Guide resins, respectively. The chamber fits within a digital heating-cooling dry bath (Thermo Fisher Scientific, Waltham, MA, United States) for temperature regulation. A schematic of the ASTHER perfusion system is available in Figure 1A The system was not transported with the eye outside of the outflow reservoir in order to prevent air currents from affecting thermal stability.

#### 2.1.1 Pig eye modified enucleation, cannulation, and perfusion

Yucatan minipigs (n = 7, 14–42 kg) were used to evaluate the ASTHER perfusion system. All animals received humane care in accordance with the Guide for the Care and Use of Laboratory Animals, and the protocol was approved by the Institutional Animal Care and Use Committee of the University of Pittsburgh. Surgical anesthesia services were provided by the Division of Laboratory Animal Resources (DLAR) at the University of Pittsburgh. Pigs were anesthetized with intramuscular ketamine (20 mg/kg) and xylazine (2 mg/kg). After intubation, anesthesia was maintained with isoflurane inhalation (1%–3%). Anesthesia depth was monitored using respiration rate, heart rate, temperature, eye drift, oxygen saturation, and blink reflex. Intramuscular methylprednisolone (1–2.5 mg/kg) and IV heparin (300–400 units/kg) were administered. Two cases deviated from the methylprednisolone administration due to drug availability, Pig 1 and Pig 7, which received no steroid and prednisone (0.08 mg/kg), respectively. If more than 1.5 h passed between initial heparin administration and the start of enucleation, a bolus of 200 U/kg heparin was administered prior to eye removal. Vascular access was established via catheterization of the femoral vein or the external carotid artery. Blood (200–500 mL) was gravity-collected and filtered (Sepacell RS-2000, Fenwal Inc., Lake Zurich, IL, United States) before priming the perfusion circuit. Dilating (NDC 42702-102-15 and NDC 70069-121-01) and intraocular pressure lowering drops (NDC 42571-141-26 or NDC 70069-051-01) were administered as part of the preparation protocol. A secondary blood draw was performed post-enucleation until presentation of hypotension or tachycardia under 5% inhaled isoflurane. Euthanasia was then immediately pursued with an intravenous dose of Euthasol at a dose of 100 mg/kg. Death was confirmed by DLAR staff using auscultation of the heart and lungs.

The modified enucleation procedure was adapted from Rousou et al. (2019) for non-craniotomy enucleation and ophthalmic artery cannulation in pigs. A periorbital skin incision was made to remove the eyelids and eyelashes, exposing the extrinsic eye muscles. Sutures were placed under the rectus muscles, and the extraocular muscles were dissected to free the eye from the orbit. The optic nerve and ophthalmic artery were severed as proximally as possible to the optic canal. Immediately after optic nerve transection, the enucleated eye was placed under a surgical microscope (OPMI Lumera 700, Zeiss, Oberkochen, Germany). The ophthalmic artery was cannulated with a 24 G ethylene tetrafluoroethylene (ETFE) 19 mm cannula (Nipro, Osaka, Japan). The cannula was secured using sutures and sealed with Dermabond (Ethicon, Somerville, NJ, United States) and Steri-Strips™ (3M Company, St. Paul, MN, United States). The eye was then transferred to the ASTHER perfusion system, where the cannulation platform was secured to the eye support chamber. The ophthalmic artery cannula was connected to the primed perfusion circuit via a wet-to-wet connection. The workflow of the modified enucleation to perfusion is visualized in Figure 1B.

When not actively undergoing imaging or electroretinography (ERG), the eyes were submerged in the outflow reservoir within the eye support chamber. The digital heating-cooling dry bath was set to 34 °C. A summary of all experiments, including pig-specific labeling and study progression, is provided in Table 1.

**TABLE 1:** Summary of experiments.

Animal	Pig 1		Pig 2		Pig 3		Pig 4	Pig 5	Pig 6	Pig 7
Experiment	OCT, AOSLO	OCT	ERG	ERG	ERG	ERG	FA	DiI	Gross fundus	Injection
Length (hrs.)	3	24	-	-	-	-	-	-	4	6
Label	P_1,OD_	P_1,OS_	P_2,OS_	P_2,OD_	P_3,OS_	P_3,OD_	P_4,OS_	P_5,OD_	P_6,OD_	P_7,OS_
Ischemia (mm.ss)	10.00	6.00	11.54	7.25	6.13	7.30	6.00	10.27	8.00	7.12
Flow (mL/min)	0.10→ 1.00*	0.1	0.10→ 0.25**	0.25	0.25	0.00	0.25	0.25	0.25	0.25
Anti-coagulant	C	C	H	H	H	--	H	H	H	H
Supplement	-	-	+	+	+	-	-	-	-	+

* Increased at 3 h of perfusion, ** increased at 10 min of perfusion, C = citrate, H = heparin, (+) included in perfusate, (-) excluded from perfusate.

### 2.2 Demonstrate retinal perfusion and evaluate retinal structure using high-resolution imaging techniques

OCT imaging was performed on enucleated eyes (n = 2) from a single pig, P_1_. In addition to OCT imaging, AOSLO imaging was performed on P_1,OD_. Experimental setup for OCT and AOSLO is visualized in Supplementary Figures S1A, B. Perfusion was initiated at 0.10 mL/min, and OCT imaging was conducted with the SPECTRALIS HRA + OCT system (Heidelberg Engineering Inc., Franklin, MA, USA) to visualize the retinal layers, retinal pigment epithelium (RPE), and choroid. AOSLO imaging was performed as described by Rui et al. (2024) to assess structure and microvascular blood flow.

For P_1,OD_, OCT imaging was performed at 0.5, 2, and 3 h, with AOSLO imaging conducted between 1 and 2 h. At the 3-h mark, flow was increased to 1.00 mL/min, and OCT imaging was repeated to evaluate the effects of increased perfusion. A 24-h perfusion was conducted on P_1,OS_ at 0.10 mL/min, with OCT imaging at 2, 8, and 20 h to assess the stability of retinal architecture under prolonged perfusion.

### 2.3 Assess retinal function by recording electroretinograms in perfused eyes

ERG recordings were performed to assess retinal function in four eyes (n = 4) from two pigs, each subjected to specific perfusion conditions.

The flow rate of P_2, OS_ was initially set to 0.10 mL/min and was raised to 0.25 mL/min after 10 min to evaluate the effects of increasing flow. P_2,OD_ and P_3,OS_ were consistently perfused at 0.25 mL/min to assess retinal function under sustained conditions. P_3,OD_ served as an ischemic control, where it was connected to the system but did not receive blood flow. To support ocular health during perfusion, a supplement (Supplementary Table S1) was added to the perfusate based on a modified version of perfusate additives used by whole body and brain perfusion publications (Vrselja et al., 2019; Andrijevic et al., 2022). The additive was not included in experiments involving imaging, as the blue coloring could confound qualitative analysis of images.

Preoperative ERGs were performed on anesthetized pigs after pupil dilation to confirm normal retinal function prior to enucleation. After enucleation, cannulation, and perfusion initiation, ERGs were continuously recorded. Between recordings, the eye was regularly irrigated with saline to maintain a tear film layer. The cannulation platform remained above the veinous outflow reservoir (not submerged).

Pre and postoperative ERGs were recorded employing a Jet contact lens electrode (Fabrinal SA, La Chaux-de-Fonds, Switzerland). Needle electrodes were employed for reference and earth electrodes. In the preoperative recordings, the reference electrode was placed at the lateral commissure of the eyelid and the earth midway between the two eyes. During postoperative recordings, ground and reference electrodes were positioned posterior to the eye in the subcutaneous fatty tissue lateral to the optic nerve, which was excised during a modified enucleation. All ERGs were recorded to a bright flash stimulus with an intensity of 10 cd/s/m^2^ under photopic conditions using the Espion E2 system (Diagnosys, Lowell, MA, United States). Signals were recorded and digitized using a sampling rate of 1 kHz and a bandpass filter of 0.312–100 Hz. The amplifiers had a fixed gain with an input range of ±0.5 V. During recordings, electrode impedance was maintained below 5 kΩ. Stimuli were generated and presented at a rate of 1 Hz using a mini ganzfeld (Colorburst, Diagnosys, Lowell, MA, United States) directly in front of the eye being tested. Signal averaging was employed with ERG averages consisting of a minimum of 50 stimuli.

### 2.4 Maintain global anatomical integrity of the eye, including vascular architecture and ocular morphology

To evaluate vascular perfusion, fluorescein angiography (FA) (n = 1, P_4,OS_) and arterial staining (n = 1, P_5,OD_) were performed. FA was conducted using the MICRON X fundus camera (Phoenix-Micron, Inc., Bend, OR, United States) following perfusion of a 1:10 dilution of fluorescein in heparinized saline at a rate of 0.25 mL/min. For arterial staining, P_5,OD_ was perfused with 1,1′-dioctadecyl-3,3,3′,3′-tetramethylindocarbocyanine perchlorate (DiI, Invitrogen, Waltham, MA, United States) using a peristaltic pump, following the protocol by Li et al. (2008). The retina was then removed, flat-mounted with Gelvatol mounting medium, and imaged. Fluorescent images were collected on the SLIDEVIEW VS200 (Evident, Tokyo, Japan) using a 0.4NA UPlanXApo objective (Olympus, Tokyo, Japan) with a 50 msec exposure time and the VS200 ASW 3.4.1 software (Evident, Tokyo, Japan).

Gross anatomy and fundus imaging were taken on a single eye (P_6,OD_) to assess structural stability and vascular perfusion at the tissue level. The eye was perfused at 0.25 mL/min. Gross and fundus images were obtained at 0, 2, and 4 h after perfusion initiation on the ASTHER system. Imaging focused on evaluating intraocular blood vessel perfusion, extraocular venous drainage, and the overall macroscopic appearance of the globe. Between imaging sessions, the eye was submerged in the temperature-controlled outflow reservoir within the eye support chamber.

### 2.5 Enable intraocular surgical procedures during perfusion

To evaluate the feasibility of an intraocular injection in a perfused eye, a subretinal injection of 100 µL of phosphate-buffered saline (PBS) was performed after 1.5 h of perfusion at 0.25 mL/min (P_7,OS_).

The eye was secured in a vertical orientation using an eye chamber attachment (Supplementary Figures S1C, D). Two 25G trocars were placed in the pars plana. A Stellaris Elite vitrectomy system (Bausch + Lomb, Ontario, Canada) was used for illumination, and PBS was injected via a PolyTip 25G/38G cannula (MedOne, Sarasota, FL, United States). Retinal structure was assessed using OCT post-injection.

### 2.6 Evaluate the applicability of the pig perfusion protocol in human tissue

To assess the potential for human eye perfusion, an organ donor eye (n = 1) was recovered via a lid-sparing enucleation using surgical loupes during routine organ donation, immediately after cross-clamp. The procurement and use of the donor eye and autologous blood were approved by the Center for Organ Recovery and Education (CORE, Pittsburgh, PA, United States) and the University of Pittsburgh Committee for Oversight of Research and Clinical Training Involving Decedents (CORID, approval No. 927). The ophthalmic artery was cannulated with an 18 G ETFE 19 mm cannula (Nipro, Osaka, Japan), secured with sutures and Dermabond, and connected to the perfusion system. The eye was immediately perfused with DiI, following the same perfusion protocol used for the pig eye.

## 3 Results

### 3.1 Develop a perfusion system capable of transport from the operating room to the laboratory without any interruption of perfusion

The ASTHER perfusion system was successfully transported between the laboratory, operating room, and multiple imaging facilities. There were no disruptions in perfusion monitoring due to the movement of the system between locations. The eye support chamber accommodated both imaging procedures and subretinal injections. Cannulation remained secure across all experiments, including during vertical repositioning, with no loss of perfusate flow or displacement of vascular access.

#### 3.1.1 Pig eye modified enucleation, cannulation, and perfusion

The modified enucleation procedure resulted in the undamaged removal of whole globes. 15–26 mm segments of the optic nerve retained enough vasculature for cannulation of the ophthalmic artery prior to branching of the central retinal artery. Surgical video of the modified enucleation process from P_5,OS_ is available in Supplementary Video S1. It is unknown how the deviation from methylprednisolone administration in Pig 1 and Pig 7 affected subsequently presented results; no metrics for inflammation or immune system response were measured during these case studies.

### 3.2 Demonstrate retinal perfusion and evaluate retinal structure using high-resolution imaging techniques

**FIGURE 2:**
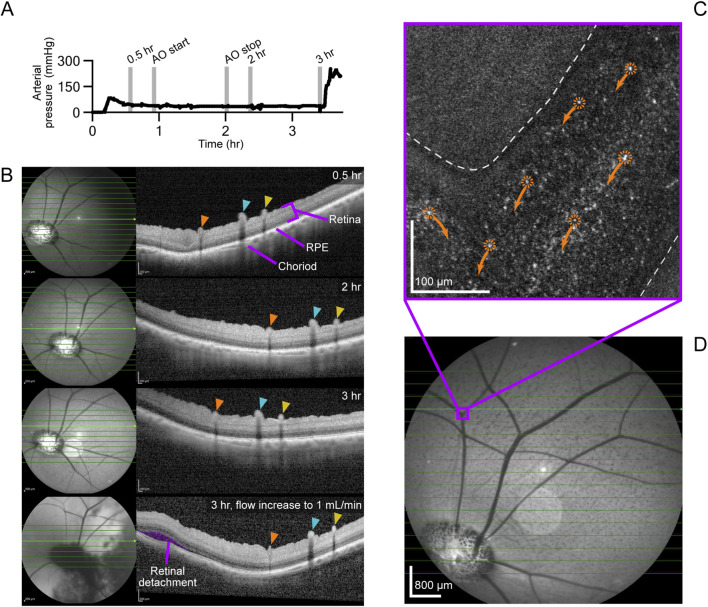
OCT and AOSLO outcomes for P_1,OD_. **(A)** Arterial pressure recordings during OCT imaging. Vertical lines indicate OCT and AOSLO imaging timepoints. **(B)** OCT and infrared fundus images at 0.5, 2, and 3-h timepoints. Orange dashed horizontal lines indicate OCT scan locations. Each colored pointer (orange, teal, yellow) identifies the same superficial retinal vessel at different time points. The purple shading indicates an area of retinal detachment. **(C)** Still image of AOSLO video, red blood cell movement direction is indicated with arrows. **(D)** Fundus image marking the location of AOSLO imaging.

**FIGURE 3:**
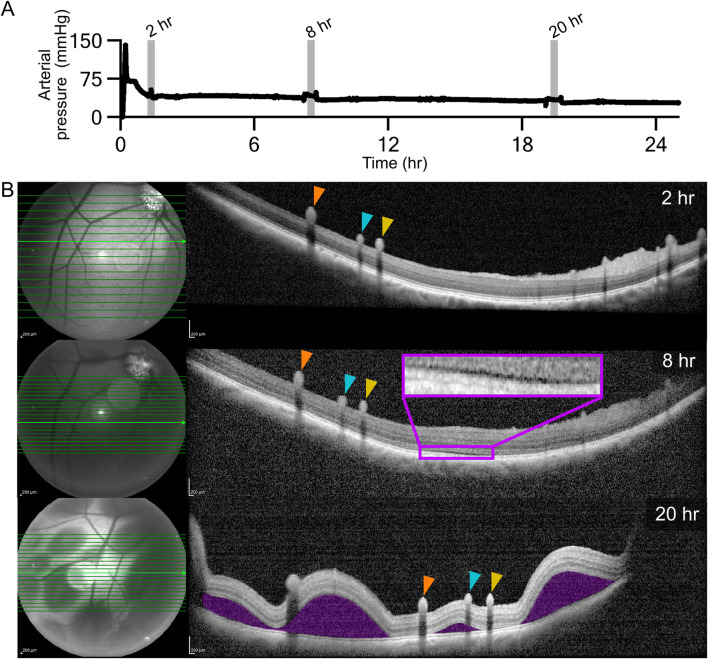
OCT images for P_1,OS_. **(A)** Arterial pressure recordings during OCT imaging. Vertical lines indicate OCT imaging timepoints. OCT **(B)** OCT and infrared fundus images at 2, 8, and 20-h timepoints. Orange dashed horizontal lines indicate OCT scan locations. Each colored pointer (orange, teal, yellow) identifies the same superficial retinal vessel at different time points. Purple blowout of the 8-h imaging shows early retinal detachment. Purple shading on the 20-h image indicates retinal detachment.

The arterial pressure waveform for P_1,OD_ is shown in Figure 2A outlining the OCT and AOSLO imaging timeline. An increase in pressure was observed 3 hours after optic nerve transection, corresponding to a rise in perfusion rate from 0.10 to 1.00 mL/min, with values increasing from 36 ± 4 mmHg to 173 ± 90 mmHg. OCT imaging revealed well-delineated retinal layers - including the ganglion cell layer, inner plexiform layer, and outer nuclear layer - without evidence of intraretinal edema, cystic changes, or serous detachment for up to 3 h at a perfusion rate of 0.10 mL/min (Figure 2B: 0.5, 2, and 3 h sub images). Confocal AOSLO images visualized individual red blood cells moving towards the optic disc through a small retinal vein, with no signs of hemorrhage, vessel wall irregularities, or vascular leakage (Figures 2C,D). A confocal AOSLO video of vascular flow is available in Supplementary Data (Supplementary Video S2). Disruptions in this supplementary video are due to drops of saline being administered to the eye in order to maintain globe hydration. Degraded image quality was observed when the cornea became dry, introducing higher-order optical aberrations. Drops of saline refreshed the tear film, returning the image quality to its stable state. The saline drop rate was controlled via a custom reservoir and roller clamp (Supplementary Figure S1B). Following an acute increase in perfusion rate to 1.00 mL/min 3 h after optic nerve transection, structural disruption was noted on OCT, including separation of the retina from the underlying RPE consistent with retinal detachment (Figures 2B 3 h. 1 mL/min sub image). The arterial pressure waveform for P_1,OS_, 24-h perfusion at 0.10 mL/min, is shown in Figure 3A with OCT imaging timepoints indicated with grey vertical bars. The average perfusion pressure over the 24-h experimental period was 35 ± 5 mmHg. OCT images showed that the retinal layers remained distinct, well-organized, and continuous, without signs of edema or cystic change for 8 h (Figure 3B 2 and 8 h sub images). A minor retinal detachment, however, was noted at the 8-h timepoint. By 20 h, widespread neurosensory detachments involving all quadrants were evident (Figure 3B 20 h sub image).

### 3.3 Assess retinal function by recording electroretinograms in perfused eyes

**FIGURE 4:**
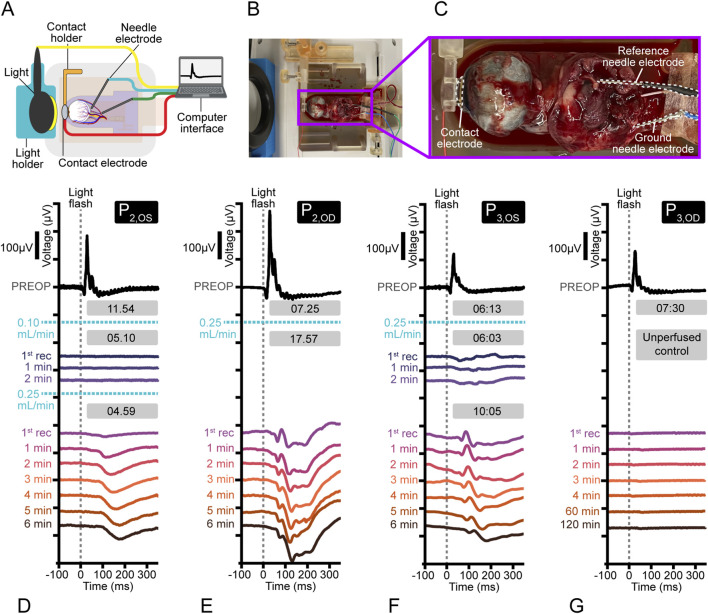
Retinal electrophysiological activity in perfused eyes after optic nerve transection. Preoperative ERGs are shown in black, and subsequent waveforms progress downward chronologically. Gray boxes indicate the elapsed time (mm:ss) either from the moment of optic nerve transection to the start of perfusion or between subsequent recordings during perfusion. Flow rates are indicated by dashed blue horizontal lines. Light stimulus occurred at time 0, marked by vertical dashed gray lines. **(A)** Schematic of ERG recording setup. **(B)** Representative image of the perfusion system setup during ERG recording. **(C)** Enlarged image showing electrode placement with the contact and needle electrodes outlined and labeled. **(D)** ERG waveforms from P_2,OS_. **(E)** ERG waveforms from P_2,OD_. **(F)** ERG waveforms from P_3,OS_. **(G)** ERG waveforms from P_3,OD_ (ischemic control).

Electrode positioning is shown in (Figures 4A–C). Preoperative ERGs were of normal morphology with well-defined a- and b-waves in all eyes prior to enucleation (Figures 4D,E, black waveforms). The mean a-wave amplitude was 27.8 ± 10.3 μV at 13.8 ± 0.5 ms, while the b-wave measured 226 ± 56.8 μV at 29 ± 1.4 ms.

ERGs with atypical morphologies were present in all eyes during *ex vivo* perfusion and varied between eyes and animals. The ischemic control eye showed no neuronal activity post enucleation. In P_2,OS_, initial recordings at 0.10 mL/min perfusion were isoelectric, showing no electrophysiological evidence of retinal activation. After increasing flow to 0.25 mL/min, ERG waveforms became evident after 4.59 min, displaying a broad negative potential at 120 ms post-stimulation. This component increased in amplitude, reaching a maximum of 48 µV over the next 7 min (Figure 4D).

The ERG responses of P_2,OD_ exhibited a negative (N1) - positive (P1) - negative (N2) complex (Figure 4E). These components measured 26 μV at 63 ms (N1), 34 μV at 80 ms (P1), and 49 μV at 110 ms (N2), respectively. Over the 7-min ERG recording period, the N1 component remained stable while the P1 component decreased in amplitude. The N2 component increased in amplitude and implicit time, reaching a maximum amplitude of 100 μV at 120 ms. ERG recordings in P_3,OS_ demonstrated a negative (N1) - positive (P1) - negative (N2) waveform 12.16 min post-optic nerve transection. These components measured 3 μV at 64 ms (N1), 2 μV at 104 ms (P1), and 2 μV at 126 ms (N2), respectively. After the eye was immersed in the venous outflow reservoir for 10.05 min and subsequently raised, ERG recordings revealed a more prominent waveform with the same N1 - P1 - N2 configuration. Over the next 7 min, the N1 and P1 components progressively decreased in amplitude. The N2 component increased in both amplitude and implicit time, ultimately reaching a peak amplitude of 45 μV at 121 ms (Figure 4F). The ischemic control eye (P_3,OD_)exhibited no detectable ERG responses for 2 h after optic nerve transection (Figure 4G). Data acquisition for the control eye was then suspended due to the absence of detectable signal.

### 3.4 Maintain global anatomical integrity of the eye, including vascular architecture and ocular morphology

**FIGURE 5:**
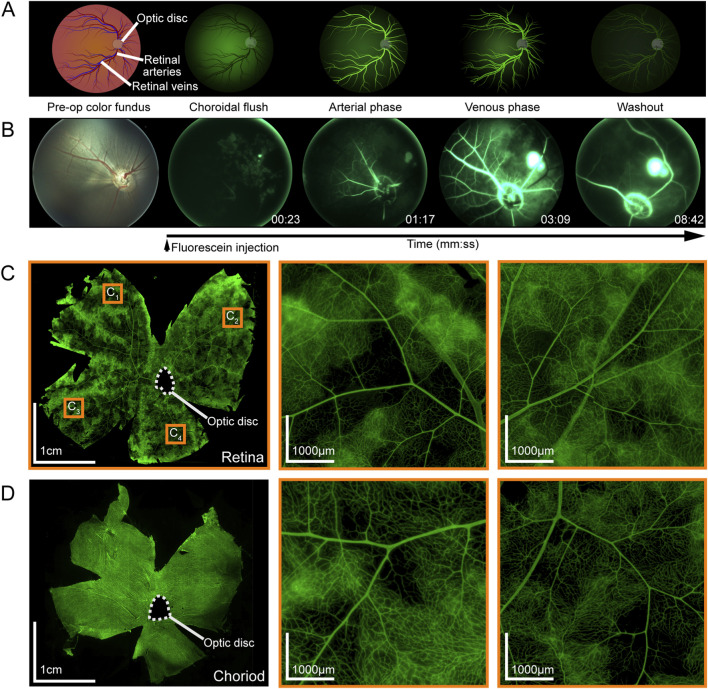
Fluorescein Angiography phases and DiI labeled flatmounts of the retina and choroid. **(A)** Diagrammatic view of typical FA phases. **(B)** Stills from FA video of P_4,OS_. Timestamp is in reference to time of injection. **(C)** Retina wholemount with vasculature visualized by DiI labeling. Regions in the superior (C_1_), temporal (C_2_), nasal (C_3_), and inferior (C_4_) quadrant at 10X show perfusion of retinal microvasculature. **(D)** Choroid wholemount with vasculature visualized by DiI labeling.

A typical FA pattern is displayed in Figure 5A. In comparison, FA imaging of P_4,OS_ demonstrated distinct choroidal and retinal vascular phases (Figure 5B) consistent with a perfusion sequence following the displayed typical FA pattern. Following fluorescein administration, the choroidal flush phase appeared first, with dye rapidly filling the choroidal vasculature. This phase presented as a diffuse, patchy fluorescence due to the fenestrated choriocapillaris, with brightness varying based on regional perfusion efficiency. As perfusion continued, the arterial phase was marked by fluorescein entry into the retinal arteries, where vessels appeared as well-defined, bright linear structures. In the venous phase, fluorescence extended into the retinal veins, initially outlining the vessel walls before achieving full luminal fluorescence. This was followed by the washout phase, during which fluorescence gradually diminished as the dye cleared from the vasculature. Timestamped FA videos at 1x and 4x speed are available in the Supplementary Videos S3, S4.

DiI stained delineating retinal arteries and veins of P_5,OD_ (Figure 5C). Choroidal flat mounts showed widespread DiI labeling throughout the choroidal vasculature (Figure 5D).

**FIGURE 6:**
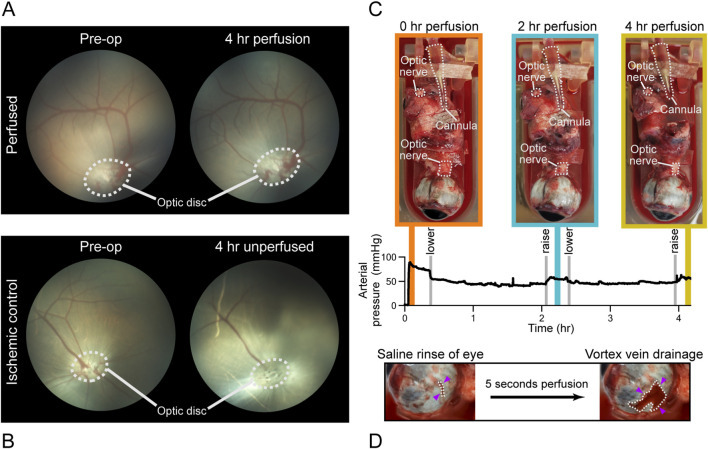
Fundus and macroscopic photograph of perfused and ischemic eyes. **(A)** Pre-operative and 4-h perfused fundus images. Optic disk is circled in white. **(B)** Pre-operative and 4-h ischemic control eye. Optic disk is circled in white. **(C)** Perfusion pressure waveform. Vertical lines indicate timepoints where eye was raised and lowered. Representative gross images at 0, 2, and 4-h timepoints. **(D)** Drainage of vortex vein after saline wash.

Fundus images of P_6,OD_ collected preoperatively and at 4-h of perfusion are qualitatively comparable and show no obvious regional losses of perfusion (Figure 6A). No signs of retinal hemorrhage, arteriovenous nicking, or occlusion were observed throughout the perfusion period. In contrast, the ischemic control eye exhibited a stagnant column of blood within a retinal vessel, accompanied by empty blood vessels observed at the 4-h timepoint (Figure 6B). Optic disc opacification can also be observed.

Perfusion of P_6,OD_ at 0.25 mL/min maintained an arterial pressure of 49 ± 9 mmHg over the 4-h perfusion period (Figure 6C). Minimal progressive scleral darkening was observed over time (Figures 6C). No evidence of corneal opacification was observed. Active bleeding was evident at multiple locations around the globe, with venous outflow originating from the vortex veins (Figure 6D).

### 3.5 Enable intraocular surgical procedures during perfusion

**FIGURE 7:**
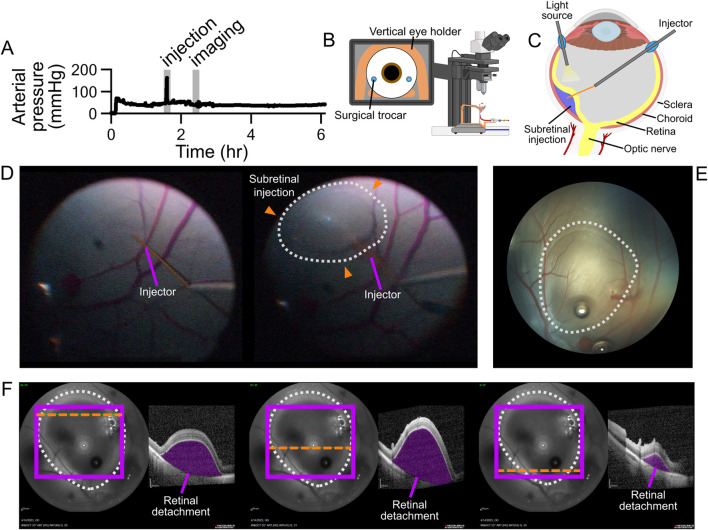
Subretinal injection in *ex vivo* perfused eye. **(A)** Pressure waveform showing pressure spike during injection. **(B)** Diagram of system setup during injection. **(C)** Surgical diagram of subretinal injection methodology. **(D)** Stills from surgical scope recording showing before and after subretinal injection retinal appearance. White dotted line shows subretinal bleb. **(E)** Fundus image of subretinal injection. White dotted line shows subretinal bleb. **(F)** OCT images of the superior, middle, and inferior portion of the subretinal injection along with infrared fundus images. Retinal detachment from injection is shaded in purple. Orange dashed horizontal lines indicate OCT scan locations.

A subretinal injection was performed on P_7,OS_ using PBS (Figure 7A. Arterial pressure was measured to be 42 ± 8 mmHg over the course of the perfusion and peaked at 168 mmHg during the injection (Figure 7B). The cannula remained in place during and after the subretinal injection on visual inspection. Fundus and OCT imaging confirmed successful sub-retinal bleb formation characterized by localized retinal detachment at the injection site (Figures 7C–E). A surgical microscope video of the injection is provided in the Supplementary Material (Supplementary Video S5).

### 3.6 Evaluate the applicability of the pig perfusion protocol in human tissue

**FIGURE 8:**
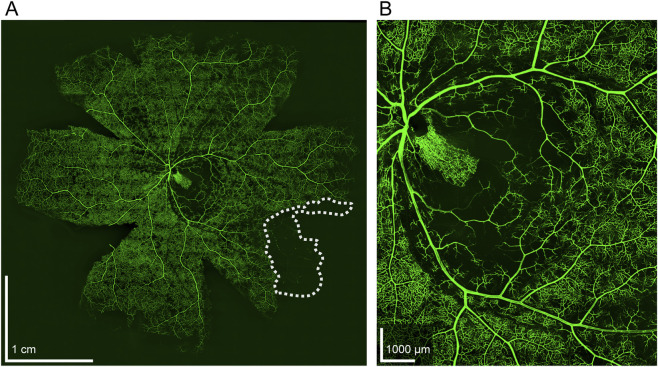
Flat mount of human retina perfused with DiI. **(A)** Entirety of the flat mount with a small region of the temporal retina showing ischemia (white dotted line). **(B)** ×10 magnification of the fovea, highlighting the cilioretinal artery branching from the posterior ciliary artery supplying the macula.

A human eye was retrieved during routine organ donation without damage to the eyelids or surrounding soft tissue. DiI administered through the ophthalmic artery cannulation site labeled downstream blood vessels, enabling visualization of perfused territories (Figure 8). A small section of the temporal retina was not perfused (Figure 8A, white dotted line). Retinal capillaries were concentrated in the macula but absent in the fovea centralis (Figure 8B).

## 4 Discussion

*Ex vivo* perfusion models have utilized a range of animal eyes–including feline, bovine, equine, ovine, canine, and murine models–to study retinal electrophysiology, pharmacokinetics, aqueous humor dynamics, and more (Niemeyer, 1973; Shahidullah et al., 2003; Shiels et al., 1999; Mains et al., 2012; Cringle and Alder, 1988; Rousou et al., 2023; Su et al., 1995; Eltanahy et al., 2023). Yucatan minipig eyes were selected as a substitute for human tissues in the pilot studies of the ASTHER system. Porcine globes are widely used in ophthalmic research and surgical training because of their anatomical similarities to human eyes–including comparable size, anterior chamber depth, and scleral thickness (Lee et al., 2006; Fallano et al., 2017; Brunette et al., 2011; Van Cruchten et al., 2017). The aforementioned animal models incorporated multiple experimental modalities such as ERG, perfusion and intraocular pressure monitoring, clinical ultrasounds, ultrasound with microbubble drug delivery, and intravenous drug delivery within a single platform. ASTHER builds upon this body of work by supporting similar multimodal experimentation in an intact globe while interfacing with clinical ophthalmological imaging devices of the 21st century.

### 4.1 Develop a perfusion system capable of transport from the operating room to the laboratory without any interruption of perfusion

A range of strategies have been used to deliver perfusate in *ex vivo* systems. Gravity-fed approaches, such as those described by Eltanahy et al. (2023), Kallab et al. (2024), and Niemeyer (2001), require manual height adjustments to maintain a constant perfusion pressure and manual agitation to prevent sedimentation (in the case of cellular perfusates). Syringe pumps provide precise delivery but are limited by reservoir volume, high shear rates, and the lack of an agitating element (in the case of cellular perfusates). Peristaltic pumps, employed in the ASTHER system, achieve consistent volumetric flow rates over a range of fluid viscosities (Mazhar et al., 2024; Abello et al., 2022), induce lower shear rates than syringe pumps, do not require continuous manual manipulation, and enable the use of a blood bag rocker (in the case of cellular perfusates). A bubble trap was added post-pump to prevent air emboli from the perfusate source. In the ASTHER system, arterial perfusion pressure was monitored using a transducer placed proximal to the cannulated artery. These design elements were informed by previous systems, such as Niemeyer (2001) model, and were aimed at maintaining stable arterial perfusion pressure and flow during experiments. To enable mobility, system power was routed through portable power stations capable of simultaneous AC input and output. This allowed for continuous power station charging while connected to wall power but uninterrupted AC output when the power stations were unplugged.

In summary, the equipment selected for the ASTHER system integrates flow regulation, arterial pressure monitoring, and an independent power supply into a cart-based system. This combination of equipment enabled the transportation of actively perfusing tissue from the operating room to the laboratory.

### 4.2 Demonstrate retinal perfusion and evaluate retinal structure using high-resolution imaging techniques

OCT imaging showed clear delineation of retinal layers, absence of edema, and no retinal detachment for up to 3 h at a flow rate of 0.10 mL/min in a single eye. Prominent superficial retinal vessels were observed, consistent with previous studies characterizing pig retinal structure using OCT (Cheng et al., 2018). AOSLO imaging visualized laminar blood flow, characterized by the lack of eddies and little to no mixing, within the retinal vasculature of an eye perfused at 0.10 mL/min for 3 h. While AOSLO is not commonplace in the clinic, it is widely used in the research setting and has been applied in human studies to visualize fine retinal structures *in vivo* (Rui et al., 2024; Zhang et al., 2015; Williams et al., 2023). Future investigations utilizing AOSLO will extend past these initial proof-of-concept images to assess the structural integrity of cellular structures as well as the immune response of the eye to the enucleation and perfusion process in long term metabolic support. To the best of the authors’ knowledge, this is the first publication containing OCT and AOSLO images of an *ex vivo* perfused eye of any large animal model (cat sized or larger).

An increase in the perfusion rate to 1.00 mL/min at the 3-h mark led to immediate retinal detachment as evidenced by OCT imaging (Figure 2B). These findings align with Niemeyer (1975) who reported that abrupt changes in perfusion induce detachment due to vacuolization and microstructural damage in the choriocapillaris. Microvasculature damage in the *ex vivo* perfused eye resulting from a rapid increase in flow was also noted by McNeish et al. (2002).

Previously published studies have maintained retinal function for up to 12 h as evidenced by the presence of electrical activity on ERGs (Niemeyer, 1973; Tseng et al., 1989; Gouras and Hoff, 1970; Thoreson and Purple, 1989) These studies do not report the condition of the retina on visual inspection (fundus, indirect ophthalmoscope, OCT, etc.), however, normal to “super”-normal ERG waveforms indicate overall attachment and function of the retina.

Prolonged perfusion at 0.10 mL/min resulted in widespread retinal detachment between the 8- and 20-h OCT imaging timepoints. This indicates a metabolic or mechanical deficit in perfusion, due to low flow rate, that resulted in retinal adhesion failure over time. Retinal adhesion *in vivo* is maintained by hydrostatic and mechanical forces, interphotoreceptor matrix adhesion, and active RPE fluid transport (Fatt and Shantinath, 1971; Tsuboi, 1987). Post-mortem studies have shown that adhesion weakens rapidly due to metabolic failure and loss of intraocular pressure regulation (Zauberman et al., 1972; Finnie et al., 2021; Ghazi and Green, 2002). Application of IOP lowering drugs prior to enucleation in this study contributed to eyeball flaccidity, which may have resulted in vitreoretinal traction and eventual retinal detachment (Oshima et al., 2015). Oxygen supplementation improves retinal adhesion, while ischemia reduces it, underlining the importance of oxidative metabolism in structural stability (Marmor and Yao, 1995; Kim et al., 1993). Given the integral role of oxygen and metabolic support in maintaining adhesion, retinal detachment illustrates that current perfusion settings are insufficient to provide the metabolic load required for normal ocular function. The lack of blood metabolites and gas data further confounds the interpretation of the presented results. Metabolite delivery is a function of the volume of metabolites present in the blood and the blood flow rate present in the vasculature. A lack of metabolite delivery could affect retinal attachment, neuronal activity, and long-term perfusion outcomes. Future studies will need to monitor blood values to determine if the blood flow rate is insufficient, the volume of metabolites in the blood is too low, or a combination of both factors. The main contribution of these case-studies shows that ASTHER serves as a high-resolution imaging platform for *ex-vivo* eye research and that using this platform can define metabolic limitations of the current experimental methods. Future work on the ASTHER system will involve determining if the current hemodynamic parameters meet the metabolic needs of the *ex-vivo* perfused eye and adjusting experimental variables in response to the results.

### 4.3 Assess retinal function by recording electroretinograms in perfused eyes

ERG studies of *ex vivo* perfused eyes have shown a cessation of retinal activity 6 or less minutes after ischemia was induced (Tazawa et al., 1972; Alder et al., 1986; Peachey et al., 1993). The ischemic control eye in this study experienced 7.30 min of ischemia prior to ERG recordings and continued ischemia during recordings. It exhibited no electrical activity as expected. Additionally, the control eye verified that neither the light source nor its potential electromagnetic field induced spurious electrical activity at the level of the recording electrodes.

Although ERG recordings showed variability in waveform shape, latency, and amplitude within and between experimental subjects there were some similarities. In all recordings, a late negativity occurring 140–160 ms after stimulation was observed. In 2 eyes (P_2,OD_ and P_3,OS_), an earlier negative-positive-negative component was observed. Some variability in waveform amplitude and latency was expected due to perfusate leakage prior to entrance of the eye from small, severed branches of the ophthalmic artery (Niemeyer, 1973). This leakage is inherent to the surgical procedure, despite attempts to cauterize visibly bleeding branches, and affects the absolute volumetric flow rate of blood that reaches the eye.

Within experimental subjects, the delayed emergence and gradual amplification of ERG responses over several minutes reflects trends observed in *ex vivo* perfused eyes of the cat, cow, and dog (Tazawa et al., 1972; Alder et al., 1986; Peachey et al., 1993; Hoff et al., 1973; Tseng et al., 1990; Seaman et al., 1965; Niemeyer and Kolder, 1983). After a period of induced ischemia, all authors noted a “recovery period” during which the ERG waveforms changed morphology and/or total amplitude before reaching pre-ischemic values. The length of these recovery periods (∼2 min–6 h) varied based on total induced ischemic time, (80 s–2 h). While the waveforms recorded from this study experienced morphological changes and increasing amplitude(s) over time, they did not appear to be returning to pre-operative shape and magnitude.

P_2,OS_ lacked electrophysical signals during perfusion at 0.10 mL/min. This supports the evidence presented in Goal 2 that a perfusion flow rate of 0.10 mL/min is insufficient to meet the metabolic demands of a functional eye. After an increase in the volumetric flow rate to 0.25 mL/min, retinal function was recorded as a broad negative potential. This pattern, decreased a- and b-wave amplitudes with prolonged latency, has also been recorded in *ex vivo* bovine eyes perfused at 20 °C (Tazawa et al., 1972). In that study, investigators noted that continued perfusion with 20 °C blood led to deterioration of electroretinographic signal, though the time-course of the deterioration was not noted. Niemeyer also noted a reduction in b-wave amplitude and increased latency in *ex vivo* perfused cat eyes with decreasing temperature (Niemeyer, 1975). In this study, the arterial perfusate was not heated and perfused eyes remained suspended above the powered-off heated venous reservoir during ERG recordings. It therefore stands to reason that the temperature of the perfused eyes dropped from venous reservoir temperature to room temperature over the course of electrophysiological data collection (Miniatur e Swine Book of Normals, 2019). The lack of temperature data and the interference of confounding factors, such as flow rate, precludes drawing conclusions on the atypical morphology of ERG waveforms in these experiments. Future work will focus on enhancing temperature control in perfused eyes to enable quantitative assessment of temperature-dependent effects.

Electrical activity recorded from eyes connected to the ASTHER system supports existing evidence of retinal function in *ex vivo* perfused eyes. However, due to a lack of temperature control, which affects neuronal electrophysiological activity, the present ERG findings are inconclusive for assessing functional preservation, highlighting a key methodological issue that must be resolved in future work.

### 4.4 Maintain global anatomical integrity of the eye, including vascular architecture and ocular morphology

FA and DiI vascular staining showed successful vascular perfusion of both the retinal and choroidal circulations. The distinct choroidal and retinal perfusion phases observed during FA were consistent with *in vivo* perfusion kinetics. Choroidal flat mounts revealed the perfusion of deeper ocular structures, confirming distribution of the perfusate within both the central retinal artery and posterior ciliary arteries. Small regions of diffuse staining were observed in portions of the retina, indicative of microcapillary leakage from endothelial dysfunction following ischemia-reperfusion injury (IRI) (Kloka et al., 2023; Zadeh et al., 2019). The ischemic period between enucleation and reperfusion of an eye is unavoidable in the experimental process. Surgeon familiarity and training, as well as reducing inefficiencies in the cannulation and reperfusion process, is paramount in reducing the length of ischemia in the experimental animal setting.

### 4.5 Enable intraocular surgical procedures during perfusion

The feasibility of intraocular surgery was demonstrated through subretinal injection of PBS. Imaging confirmed the expected post-injection retinal detachment. These findings illustrate that the ASTHER system can accommodate intraocular manipulations such as subretinal injection, a procedure commonly employed in both experimental and clinical retinal therapies. Perfusion pressure spiked to 168 mmHg over the course of the injection, though the cannula remained secure. During the injection, elevated resistance within the retinal vasculature could have led to a greater volume of blood escaping through micro-arterial bleeds located anterior to the scleral surface. This would reduce the volumetric flow rate delivered directly to the eye during the injection period. Effects of the sub-retinal bleb and spike in perfusion pressure were not evaluated beyond the immediate post-injection period. Future work will include repeated evaluations of bleb size and retinal detachment over time.

### 4.6 Evaluate the applicability of the pig perfusion protocol in human tissue

This singular case study of the utilization of *ex-vivo* perfusion with human tissues serves as proof of technical feasibility. DiI imaging confirmed widespread retinal perfusion of a single perfused human eye, though a small temporal region remained ischemic. Possible explanations for the ischemic region include, but are not limited to, emboli ingestion, intravascular clot formation, improper circuit degassing or bubble trap failure resulting in ischemia secondary to gas ingestion, insufficient pressure generation failing to open the vascular region, and individual vascular anatomical variants. The qualitative readout from DiI imaging is insufficient to draw conclusions as to the exact cause for the presented case. The presence of a cilioretinal artery, a feature found in approximately 6.9%–49.5% of individuals, was found in the donor eye (Schneider et al., 2021). For the donation of human tissues, streamlined communication between organ procurement organizations, surgical teams, and experimentalists is required to minimize the ischemic gap the eye experiences between aortic cross clamp, enucleation, transportation, and re-perfusion. The minimal availability and unpredictability of tissue donation serves as a technical hurdle for human studies; improving procurement center relations and patient/family education are priorities for future experimentation.

### 4.7 Final comments

The experiments reported in this document serve as proof of concepts for the mobile design and the adaptation of the ASTHER system to clinical evaluation (fundus, OCT, ERG, DiI, and FA). Additional experiments will be required to draw conclusions about the efficiency, repeatability, or quality of data produced using the ASTHER system. Future refinement of system, such as improved temperature control, improved eye lubrication techniques, evaluation of the effects of changing perfusate flow rate, measurement of eye metabolic function, and increased sample sizes, will aim to better quantify the condition of arterially perfused *ex vivo* eyes and evaluate experimental repeatability.

## Data Availability

The original contributions presented in the study are included in the article/Supplementary Material, further inquiries can be directed to the corresponding author.
