# Supplementary material for: A mobile system for whole eye perfusion supporting retinal function and surgery
Source: Front Bioeng Biotechnol. 2026 Jan 16;13:1699876. doi: 10.3389/fbioe.2025.1699876 (PMC12856301; doi:10.3389/fbioe.2025.1699876)
Supplement: Supplementary file 2 [file Table1.docx]

Supplementary Material

## Supplementary Figures

Supplementary Table 1: Components of ASTHER blood supplement. Shared perfusate agents are used in both Andrijevic et al. 2022 and Vrselja et al. 2019.

| **Cytoprotective agents** | **M.W. (g/mole)** | **Concentration (mg/L)** |
| --- | --- | --- |
| Hexahydro-2-imino-1H-thieno[3,4-d]imidazole-4-pentanoic acid (2-Iminobiotin)** | 243 | 0.303 |
| 5-(1H-Indol-3-ylmethyl)-3-methyl-2-thioxo-4-Imidazolidinone (Necrostatin-1)** | 259 | 1.515 |
| Sodium 3-Hydroxybutyric Acid** | 126 | 40 |
| Glutathione Monoethyl Ester ** | 335 | 1.7 |
| Minocycline** | 494 | 1.7 |
| Lamotrigine** | 256 | 1.14 |
| 5-(2,6-Difluorophenoxy)-3-[[3-methyl-1-oxo-2-[(2-quinolinylcarbonyl)amino]butyl]amino]-4-oxo-pentanoic acid hydrate (QVD-Oph)** | 513 | 0.758 |
| Methylene Blue** | 320 | 1.515 |
| z-VAD-FMK | 467.49 | 2.3375 |
| Ferrostatin-1 | 262.35 | 1.3118 |
| Disufenton sodium (NXY-059)* | 381 | 0.38 |
| **Antibiotics and anti-inflammatory** |  |  |
| Ceftriaxone** | 661 | 121.212 |
| Dexamethasone** | 392 | 10.606 |
| **Metabolic compounds** |  |  |
| D-(+)-Glucose* | 180 | 1255.86 |
| Sodium Pyruvate* | 110 | 31.427 |
|  |  |  |
| KEY |  |  |
| *Vrselja et al. 2019 perfusate agents |  |  |
| **Shared perfusate agents |  |  |
|  |  |  |

**Supplementary Figure 1: (A) Eye on cannulation platform suspended above the eye support chamber for use with OCT. A custom reservoir and roller clamp controlled saline drips used to maintain corneal hydration. (B) Eye on cannulation platform suspended above the eye support chamber resting on a 3D-printed platform used for AOSLO imaging. The custom reservoir and roller clamp also controlled corneal hydration in this setting. (C) Eye chamber attachment used to vertically orient the eye during surgical procedures. (D) Eye in vertical orientation being surgically manipulated**.

**Supplementary Video 1: Surgical video of the modified enucleation process from P_5,OS_. Video was recorded using the OPMI Lumera 700 surgical microscope from Zeiss.**

**Supplementary Video 2: Confocal AOSLO video of vascular flow. Disruptions in this supplementary video are due to “blinks” – drops of saline administered to the eye to maintain corneal hydration controlled via a custom reservoir and roller clamp.**

**Supplementary Video 3: Timestamped video of fluorescein angiography. Video was recorded using MICRON X fundus camera.**

**Supplementary Video 4: Supplementary Video 3 presented at four times speed to enhance perceptual salience of the progression of fluorescein through the retinal vasculature.**

**Supplementary Video 5: Surgical video of subretinal injection performed in ex-vivo perfused eye.**
