# Supplementary figures and images for: A mobile system for whole eye perfusion supporting retinal function and surgery
Source: Front Bioeng Biotechnol. 2026 Jan 16;13:1699876. doi: 10.3389/fbioe.2025.1699876 (PMC12856301; doi:10.3389/fbioe.2025.1699876)

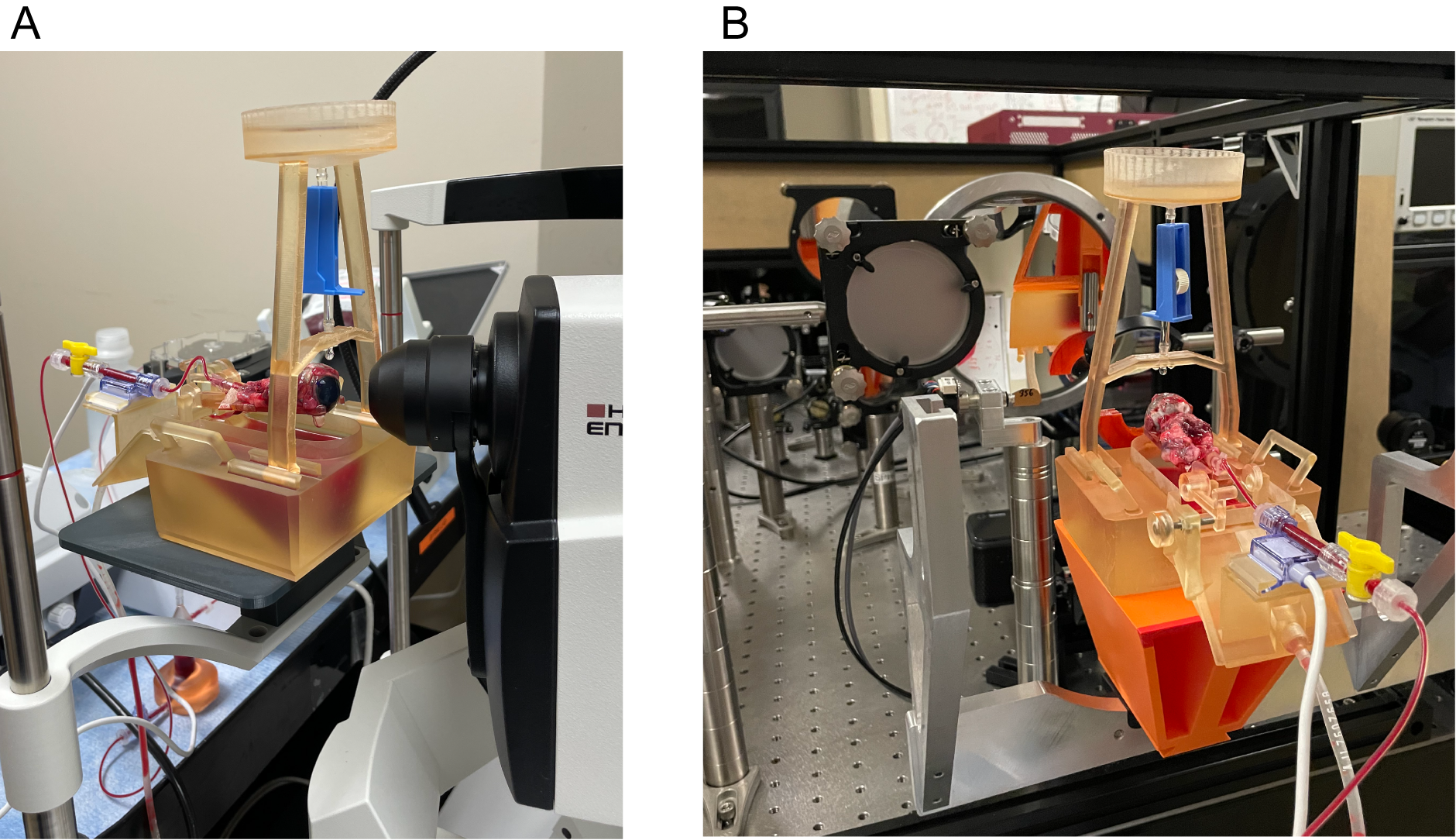

Supplement: Supplementary file 3 [file Image1.tif]
